# Dimorphic glioblastoma with glial and epithelioid phenotypes: Clonal evolution and immune selection

**DOI:** 10.3389/fneur.2022.1017087

**Published:** 2023-01-10

**Authors:** Mark Willy L. Mondia, Michael A. Kritselis, John E. Donahue, Heinrich Elinzano, Sasmit Sarangi, David Bryant, Marzia Capelletti, W. Michael Korn, Esther Yu, Sherry Yan, Steven A. Toms, Eric T. Wong

**Affiliations:** ^1^Department of Neurosciences, College of Medicine and Philippine General Hospital, University of the Philippines Manila, Manila, Philippines; ^2^Department of Pathology and Laboratory Medicine, The Warren Alpert Medical School of Brown University, Rhode Island Hospital, Providence, RI, United States; ^3^Department of Neurology, The Warren Alpert Medical School of Brown University, Rhode Island Hospital, Providence, RI, United States; ^4^Caris Life Sciences, Irving, TX, United States; ^5^Department of Radiation Oncology, The Warren Alpert Medical School of Brown University, Rhode Island Hospital, Providence, RI, United States; ^6^Department of Neurosurgery, The Warren Alpert Medical School of Brown University, Rhode Island Hospital, Providence, RI, United States; ^7^Division of Hematology/Oncology, Department of Medicine, The Warren Alpert Medical School of Brown University, Rhode Island Hospital, Providence, RI, United States

**Keywords:** epithelioid glioblastoma, exome sequencing, immune profiling glioblastoma with glial and epithelioid phenotypes, malignant glioma, clonal evaluation

## Abstract

**Purpose:**

Epithelioid glioblastoma is an unusual histologic variant of malignant glioma. The present study investigates both the genomic and transcriptomic determinants that may promote the development of this tumor.

**Methods:**

Whole-exome sequencing (WES) and whole-transcriptome sequencing (WTS) were performed on an epithelioid glioblastoma, along with a specific bioinformatic pipeline to generate electronic karyotyping and investigate the tumor immune microenvironment. Microdissected sections containing typical glioblastoma features and epithelioid morphology were analyzed separately using the same methodologies.

**Results:**

An epithelioid glioblastoma, with immunopositivity for GFAP, Olig-2, and ATRX but negative for IDH-1 and p53, was identified. The tumor cell content from microdissection was estimated to be 85–90% for both histologic tumor components. WES revealed that both glioma and epithelioid sections contained identical point mutations in *PTEN, RB1, TERT* promoter, and *TP53*. Electronic karyotype analysis also revealed similar chromosomal copy number alterations, but the epithelioid component showed additional abnormalities that were not found in the glioblastoma component. The tumor immune microenvironments were strikingly different and WTS revealed high levels of transcripts from myeloid cells as well as M1 and M2 macrophages in the glioma section, while transcripts from CD4+ lymphocytes and NK cells predominated in the epithelioid section.

**Conclusion:**

Epithelioid glioblastoma may be genomically more unstable and oncogenically more advanced, harboring an increased number of mutations and karyotype abnormalities, compared to typical glioblastomas. The tumor immune microenvironment is also different.

## Introduction

Glioblastoma is the most frequent malignant glial neoplasm in adults with a median survival near 21 months when treated with radiation, temozolomide, and tumor treating fields (1). The pathological classification has been updated recently accounting for the molecular heterogeneity of this tumor (2). However, epithelioid glioblastoma is still an outlier that is difficult to categorize based on the new WHO classification scheme. It often coexists with glioblastoma or other types of diffuse glioma (3–6). More importantly, over half of the cases harbor a *BRAF V600E* mutation making them potentially treatable with V600E-directed targeted therapy (4, 7, 8).

We described molecular findings from a case of newly diagnosed glioblastoma possessing both glial and epithelioid histologies and performed whole-exome sequencing (WES) and whole-transcriptome sequencing (WTS) of both components separately. The genomic signatures were similar in the two components and both harbored identical mutations in *PTEN, RB1, TERT* promoter, and *TP53*. However, compared to the glioblastoma background, the epithelioid component possessed additional copy number alterations as well as different and unique immune cells infiltration by CD4+ lymphocytes and NK cells, strongly supporting the hypothesis that the epithelioid component of the tumor evolved from a background of glioblastoma and could have been arisen by increased tumor instability or selected by specific tumor microenvironment niche.

## Materials and methods

### Patient and tumor tissue

**Figure 1:**
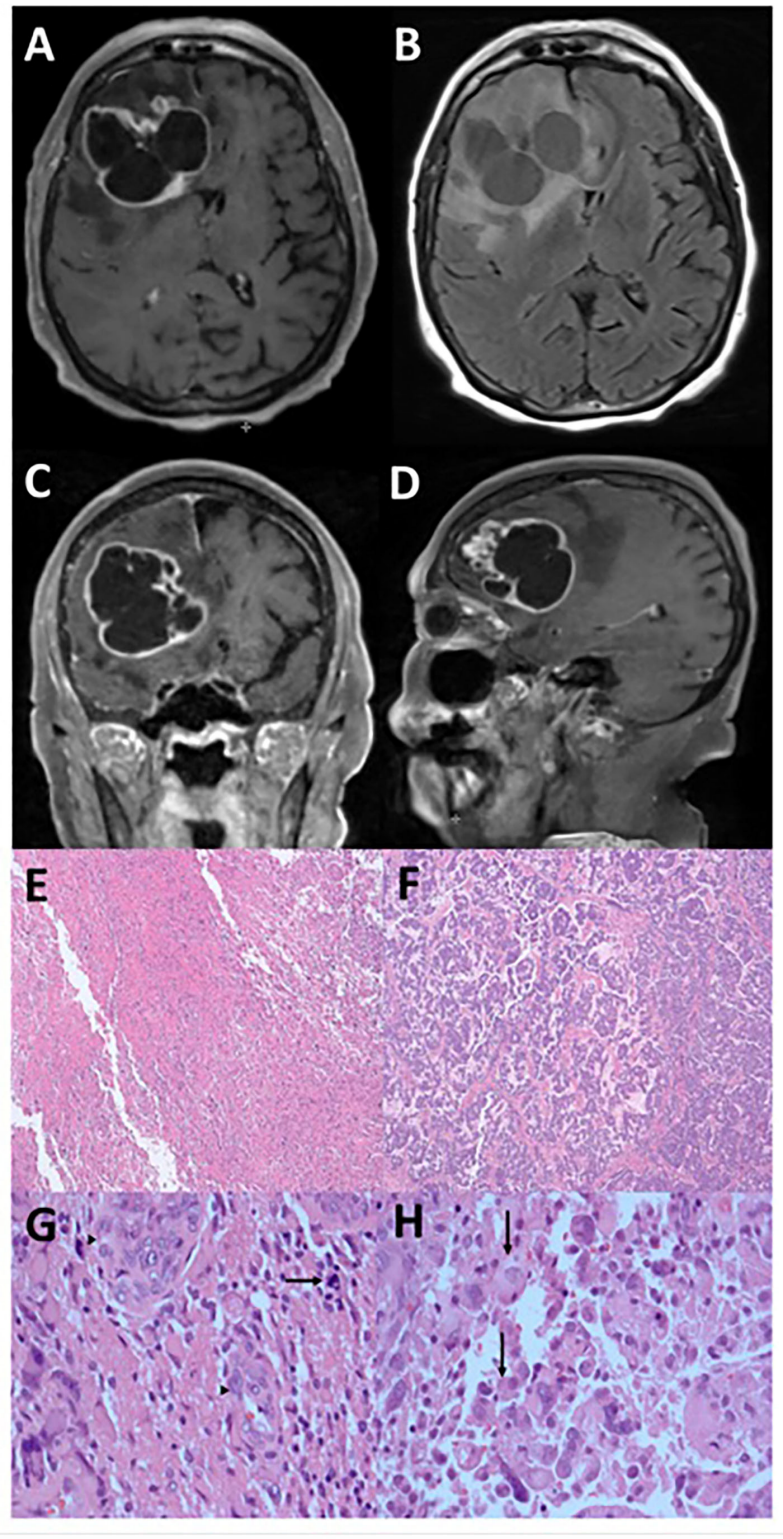
Radiographic and microscopic features of epithelioid glioblastoma. This tumor has an irregular, multi-cystic enhancement seen on post-gadolinium T1-weight MRI sequence in the axial **(A)**, coronal **(C)**, and sagittal **(D)** orientations, accompanied by surrounding parenchymal infiltration and cerebral edema **(B)**. Low magnification microscopy reveals distinct glioblastoma **(E)** and epithelioid **(F)** components (hematoxylin and eosin, 4X). Under high magnification (hematoxylin and eosin, 400X), tumor cells in the glioblastoma component display significant atypia and no distinct cell borders, together with mitosis (arrow) and endothelial proliferation (arrowheads) **(G)**. In contrast, the epithelioid component contains abundant eosinophilic cytoplasm and clear cell borders (arrows) **(H)**.

An 86-year-old woman, with a history of triple-positive (estrogen, progesterone, and Her2 receptors) invasive ductal carcinoma of the left breast (stage pT1bN0) and treated successfully with resection and external beam radiotherapy only, experienced paroxysmal seizures. A gadolinium-enhanced head MRI demonstrated a multi-cystic heterogeneously enhancing mass in the right frontal brain, measured at 5.1 x 6.2 x 6.2 cm and accompanied by 8 mm of midline shift (Figures 1A–D). CT of the torso was negative for malignancy. She subsequently underwent gross total resection of the tumor. Routine pathology studies were performed along with multi-omic comprehensive profiling by Caris Life Sciences (Irving, Texas). Adequate tumor content from formalin-fixed paraffin-embedded (FFPE) specimens was first reviewed by a board-certified pathologist at Caris, and then micro-dissected sections from different morphologies within the same tumor were performed according to pre-specified standard procedures.

### Immunohistochemistry analysis

Immunohistochemistry (IHC) was performed on full FFPE sections at the hospital and Caris. Slides were stained using automated staining techniques, as per the manufacturer's instructions, and were optimized and validated per Clinical Laboratory Improvement Amendments (CLIA) and International Organization for Standardization (ISO) requirements. Staining was scored for intensity (0 = no staining; 1+ = weak staining; 2+ = moderate staining; 3+ = strong staining) and staining percentage (0–100%). Results were categorized as positive or negative by defined thresholds specific to each marker based on published clinical literature that associates biomarker status with patient responses to therapeutic agents. A board-certified pathologist evaluated all IHC results independently. The primary antibody for programmed death-ligand 1 (PD-L1) was SP142, and the staining was regarded as positive if its intensity on the membrane of the tumor cells was ≥2+, and the percentage of positively stained cells was >5%.

### Next-generation sequencing: DNA analysis

Whole-exome sequencing was performed on genomic DNA isolated from a microdissected, FFPE tumor sample using the NovaSeq6000 sequencers (Illumina, Inc., San Diego, CA). A hybrid pull-down panel of baits designed to enrich for more than 700 clinically relevant genes at high coverage and high read-depth was used, along with another panel designed to enrich for an additional >20,000 genes at a lower depth. A 500-Mb SNP backbone panel (Agilent Technologies, Santa Clara, CA) was added to assist with gene amplification and deletion measurements. The performance of the WES assay was validated for sequencing variants, copy number alteration, tumor mutational burden, and microsatellite instability. The test was validated to 50 ng of input and has a positive predictive value (PPV) of 0.99 against a previously validated next-generation sequencing (NGS) assay. WES can detect variants with tumor nuclei as low as 20% and a variance of 5% variant frequency with an average depth of at least 500x. This test has a sensitivity to detect as low as 10% of the cell population containing a mutation in all exons from the high read-depth clinical genes and 99% of all exons in the 20 K whole-exome regions. Matched normal tissue was not sequenced.

### Next-generation sequencing: RNA analysis

Whole-transcriptome sequencing was performed on the same micro-dissected FFPE tumor sample. Gene fusion detection was performed on mRNA isolated from each FFPE tumor sample using the same NovaSeq platform (Illumina, Inc., San Diego, CA) and SureSelect Human All Exon V7 bait panel (Agilent Technologies, Santa Clara, CA). RNA FFPE tissue extraction kit (Qiagen, Hilden, Germany) was used for extraction, and the RNA quality and quantity were determined using the TapeStation (Agilent, Santa Clara, CA). Biotinylated RNA baits were hybridized to the synthesized and purified cDNA targets, and the bait–target complexes were amplified in a post-capture polymerase chain reaction. The resultant libraries were quantified and normalized, and the pooled libraries were denatured, diluted, and sequenced; the reference genome used was GRCh37/hg19, and analytical validation of this test demonstrated ≥97% positive percent agreement (PPA), ≥99% negative percent agreement (NPA), and ≥99% overall percent agreement (OPA) with a validated comparator method. The expression level of some genes selected by their importance in high-grade glioma was compared to a Caris' cohort of high-grade gliomas and expressed in percentile. Genes with an expression between the 20th and 80th percentile are considered within the variability of natural expression in cancer cells, but those with expression above the 80th or below the 20th percentile are considered noteworthy outliers.

### Electronic karyotyping

Somatic structural variants such as whole or partial chromosome duplications or deletions are important for cancer development and progression. Copy-number alterations (CNAs) associated with human cancers range from chromosomal aneuploidy to microduplication and microdeletion syndromes and include smaller structural variants (SVs) that affect single genes and exons. In traditional cytogenetics, the comprehensive analysis of all structural aberrations in a given sample requires chromosomal karyotyping, fluorescence *in situ* hybridization, and CNV microarrays. However, NGS sequencing allows the visualization of cytogenetic aberrations across the entire genome. The log2 ratio is the moving average of the copy number of the chromosome, at roughly 1 Mb resolution. The copy number is a smoothed non-log representation of the estimated ploidy across arm-level parts of the genome.

### Tumor immune cell content

The immune context of the tumor microenvironment may offer insight into the development and clonal progression of glioblastoma (9). A computational pipeline for the quantification of the tumor immune context from human RNA-seq data (quanTIseq) was used to measure the fraction of infiltrating immune cells within the tumor (10). The cell types quantified include B cells, classically activated macrophages (M1), alternatively activated macrophages (M2), monocytes, neutrophils, natural killer (NK) cells, non-regulatory CD4+ T cells, CD8+ T cells, regulatory CD4+ T cells (Tregs), dendritic cells, and other unclassified cells.

## Results

### Glial and epithelioid phenotypes were found in the glioblastoma

The histologic features of the tumor consisted of two populations of cells, with one section having the typical glioblastoma morphology (Figures 1E, G) accompanied by endothelial proliferation and pseudopalisading necrosis, while the other consisting of larger cells with epithelioid morphology (Figures 1F, H). In both components, immunohistochemistry analysis showed positivity in GFAP, Olig-2, and ATRX but negative for IDH-1 and p53 indicating wild type for both markers. The Ki-67 proliferation rate was ~40–50%. A molecular study of the DNA repair enzyme O^6^-methylguanine-DNA methyltransferase (MGMT) showed that the promoter region was methylated. Therefore, the histology is consistent with a grade 4 *IDH-1* wild-type glioblastoma typically found in the elderly population, but her prognosis may be better than average due to the methylated promoter of *MGMT*.

### Next-generation sequencing revealed similar genomic signatures

Whole-exome sequencing and whole-transcriptome sequencing were performed on both components of the tumor. Tumor content was assessed at 90% of tumor cells in the typical glioma section and 85% in the epithelioid section. The two components of the tumor revealed similar genomic signatures and pathogenic variants, and both harbored identical point mutations in *PTEN* c.610C>G, RB1 c.55G>T, and *TERT* promoter c.146C>T, as well as *TP53* c.796G>A and c.586C>T. The variant allele frequencies (VAFs) of these common mutations were much higher in the epithelioid component strongly supporting the hypothesis of a morphological change, clonal selection, and progression of the glioblastoma to epithelioid cells. Two alterations were uniquely identified in the epithelioid component: a non-sense mutation in *TAF1* (c.3619C>T, p.R1207^*^) and a missense mutation in *GATA6* (c.874 G>T, p.G292C) genes with a variant allele frequency of 24 and 49%, respectively. A search of the Catalog Of Somatic Mutations In Cancer (COSMIC) database (www.cancer.sanger.ac.uk/cosmic/gene/samples) did not reveal pathogenicity from these two mutations. However, analysis of The Cancer Genome Atlas (TCGA) dataset revealed that *TAF1* and *TP53* may be in the same driver pathway for glioblastoma development (11).

### Karyotype analysis revealed evidence of clonal evolution

**Figure 2:**
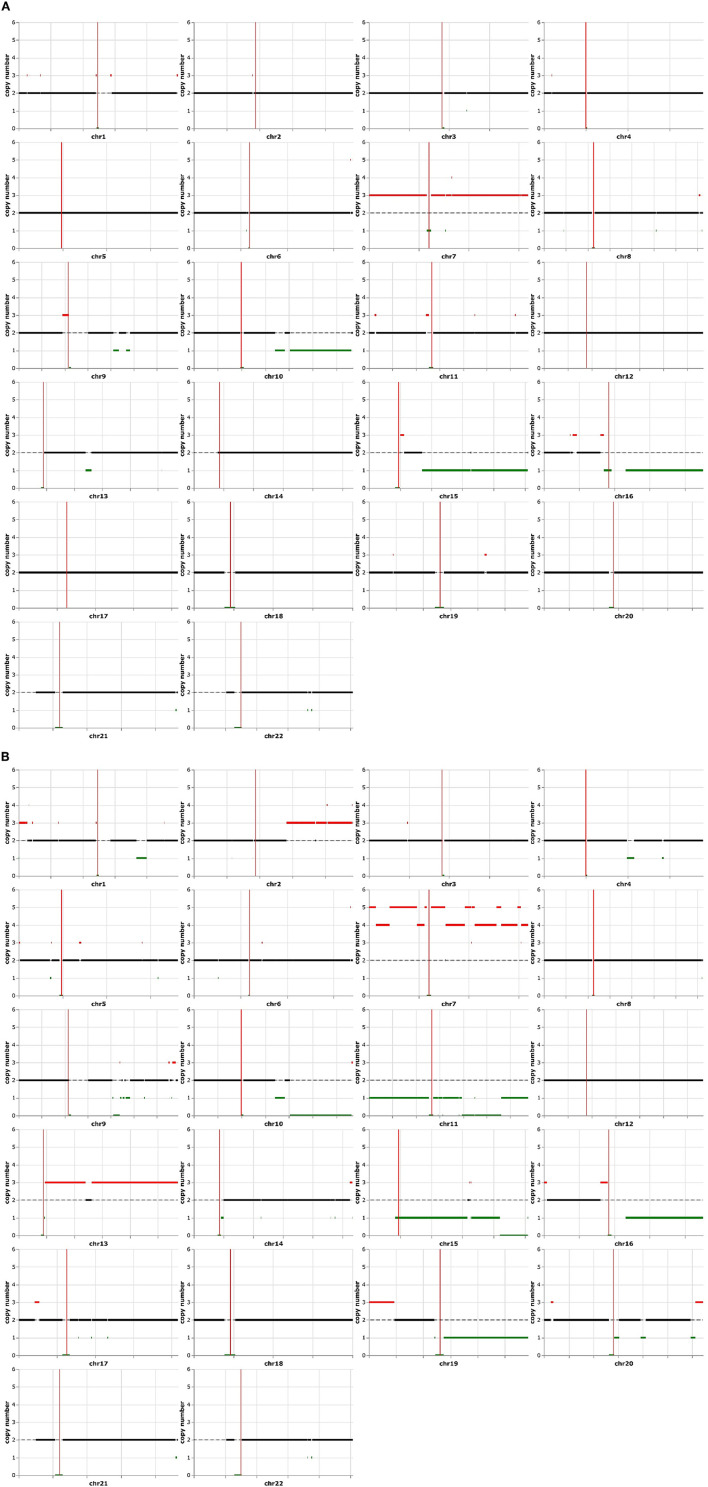
Electronic karyotype analysis of typical glioblastoma and epithelioid components. Both sections have the amplification of chromosome 7 and partial deletion of chromosomes 10q, 15q, and 16q **(A)**. The epithelioid area contained additional karyotype abnormalities, consisting of a higher copy number of chromosome 7q, amplifications of chromosomes 1p, 13q, 16p, and 19q, as well as the deletion of chromosomes 1q, 11p, 11q, 19q, and 20q **(B)**.

Electronic karyotype analysis revealed that both components contained similar copy number alterations involving extra copies of chromosome 7, a deletion of chromosome 16q, and a partial deletion of chromosomes 10q and 15q (Figures 2A, B), consistent with the molecular data showing copy number gain of *EGFR* on chromosome 7 (7p11.2) and copy number loss or deletion of *PTEN* on chromosome 10 (10q23.31). Interestingly, these shared alterations were more pronounced in the epithelioid component, and additional copy number changes were acquired including the gain of part of chromosome 2q and chromosome 13, loss of chromosomes 11 and 19q, as well as other more focal alterations in various chromosomes (Figure 2B). These findings suggest that the epithelioid section most likely evolved or differentiated from the glioblastoma due to increased genomic instability.

### Genotyping and whole-transcriptome sequencing revealed key differences in the immune cell population

Human leukocyte antigen (HLA) genotyping and WTS were performed to query potential differences in immune cell populations within the two sections. No differences were found in MHC class I and class II antigens (Supplementary Table 1). Whole-transcriptome analysis was performed for both histologic components of the tumor and compared to a cohort of glioblastoma expression data from the database at Caris. The expression of every single gene was then reported in percentiles as well as transcripts per million. We then compared transcripts expressed in the highest quintile in one of the components to the lowest quintile in the other and identified SDHD, one of the four subunits of succinate dehydrogenase, a mitochondrial enzyme involved in two essential metabolic processes such as the electron transport chain and the Krebs cycle, and CD274 (PD-L1) with higher expression in the glial than the epithelioid population (Table 1). We did not find any *vice versa* that was highly expressed in the epithelioid but not in the glioblastoma population. In addition, the epithelioid component showed a higher expression of topoisomerase 1 (TOP1) which may suggest increased susceptibility to genotoxic agents (12). Ultimately, analysis of the immune cell transcriptome revealed significant differences in the composition of immune cell populations. In particular, high levels of myeloid cells (80th percentile), as well as M1 and M2 macrophages (94th and 97th percentiles, respectively), were found in the glioblastoma component, while CD4+ lymphocytes and NK cells (100th and 98th percentiles, respectively) were elevated in the epithelioid component (Table 2). Interestingly, B cells were higher in the glioblastoma compared to the epithelioid component (89 vs. 20th percentile) (Table 2). Together, these findings suggest that these two cellular subpopulations present in the tumor appear to be under different immunologic and reprogramming selection pressures.

**Table 1:** Gene expression by whole-transcriptome sequencing.

	**Glioma section**		**Epithelioid section**	
**Gene**	**Transcript per million**	**Percentile in cancer type**	**Transcript per million**	**Percentile in cancer type**
SDHD^a^	28.827100	87.0%	8.000580	16.0%
CD274^b^	7.870500	80.0%	1.341030	18.0%

a Succinate dehydrogenase complex subunit D.

b CD274 is also known as programmed death-ligand 1 (PD-L1).

Significant differences were seen in SDHD and CD274.

Data on other transcripts are available in Supplementary Table 1.

**Table 2:** Tumor immune cell content by immune cell whole-transcriptome sequencing.

	**Glioma section**		**Epithelioid section**	
**Cell type**	**Percentage in sample**	**Percentile in cancer type**	**Percentage in sample**	**Percentile in cancer type**
B cell	9.5%	88.5%	5.7%	20.0%
Macrophage M1	10.1%	93.5%	0.0%	0.0%
Macrophage M2	11.5%	97.0%	0.0%	0.0%
Monocyte	0.0%	0.0%	21.9%	99.5%
Neutrophil	0.0%	0.0%	0.0%	0.0%
NK cell	5.9%	55.5%	12.3%	99.5%
T cell CD4+	0.0%	0.0%	1.7%	97.5%
T cell CD8+	0.0%	0.0%	0.0%	0.0%
T cell regulatory (Tregs)	0.0%	0.0%	0.0%	0.0%
Myeloid dendritic cell	11.1%	80.0%	0.0%	0.0%
Uncharacterized cell	51.9%	3.5%	58.4%	11.5%

## Discussion

Epithelioid glioblastomas are characterized by larger cells with epithelioid morphology. They may be found together with other glioma histology including low-grade astrocytoma (3, 4) and pleomorphic xanthoastrocytoma (5), and gliosarcoma (6). In our patient, the histopathology consists of distinct regions of typical glioblastoma features and atypical epithelioid morphologies. However, both are positive for GFAP and have similar genomic signatures, consisting of identical point mutations in *PTEN, RB1, TERT* promoter, and *TP53*. Therefore, these two components most likely arise from a common glioblastoma progenitor cell. Notably, the epithelioid component contains unique karyotype changes, consisting of a higher copy number of chromosome 7q, amplifications of chromosomes 1p, 13q, 16p, and 19q, as well as the deletion of chromosomes 1q, 11p, 11q, 19q, and 20q, in addition to all of the karyotype abnormalities found in the typical glioblastoma component. We believe that the changes are not from differences in tumor content due to the strict quality control process before analysis. Therefore, these additional chromosomal copy number abnormalities strongly indicate that the epithelioid component is further along in the evolutionary process compared to the typical glioblastoma cells in the background. Furthermore, Hatae et al. (13) described a case of clonal evolution during the treatment of epithelioid glioblastoma. Compared to the initial grossly resected tumor specimen, the recurrent tumor possessed loss of heterozygosity of 1p, 10q, 17q, and 19q chromosomes, as well as a new C228T *TERT* promoter mutation, while the status of wild-type IDH1/IDH2 and mutant BRAF V600E was unchanged (13). Collectively, these data suggest that epithelioid glioblastoma may have increased genetic instability, and it is prone to develop over time additional genetic mutations, copy number alteration changes, or both. Future single-cell analysis may reveal subtle differences in individual tumor cells, which may be drivers of subsequent oncogenic evolution.

Next-generation sequencing revealed a missense mutation in *GATA6* and a non-sense mutation in *TAF1* genes in the epithelioid component. First, GATA6 is a transcription factor that has broad functional significance for epithelial differentiation during development, and it is highly expressed in astrocytes, neurons, and endothelial cells (14, 15). GATA6 is also a tumor suppressor, and deletions or mutations are frequently found in high-grade astrocytic tumors (16). More importantly, gene knock-in experiments reduce their tumorigenic potential and vascular endothelial growth factor secretion (16). Although the specific mutation identified in this patient's epithelioid component has not been previously described, predictor software analysis (Polyphen-2, SIFT) suggests that this *GATA6* mutation may have a disruptive effect on transcription and could have de-differentiated the tumor into a more primitive and aggressive form. Second, TAF1 or TATA-box binding protein-associated factor 1 is the largest subunit of the transcription factor TFIID, which plays a role in neurodevelopment and p53-dependent DNA damage response. Patients with a missense mutation of *TAF1* suffer from X-linked intellectual disability (17). TAF1 also phosphorylates p53, stabilizing it and mediating cell cycle arrest in response to DNA damage (18, 19). Therefore, it is possible that a non-sense mutation would affect DNA damage response in the central nervous system, resulting in unregulated G1 progression and accumulation of additional somatic mutations.

The reduced expression of the *SDHD* gene, as well as other genes encoding the four subunits of succinate dehydrogenase, a mitochondrial enzyme involved in two essential metabolic processes, suggests possible metabolic reprogramming (20). Recent studies have shown that this kind of metabolic adaptation in glioblastoma allows invasive cells to generate the energy necessary to promote tumor invasion using available nutrients and oxygen within the new tumor microenvironment (21). Concomitantly, glioblastoma cells adapt to avoid detection and escape from the host immune system as highlighted by the reduced expression of CD274 during the morphological change in epithelioid phenotype (22).

The variations in morphology may be a result of the genetic heterogeneity of tumor cells and the selective pressure exerted by the host's immune system. First, tumor cells can exhibit regional heterogeneity in their molecular makeup within a glioblastoma. Snuderl et al. (23) found diverse amplification patterns of receptor tyrosine kinase genes in different regions of the tumor leading to intra-tumoral heterogeneity. Single-cell RNA sequencing of individual human glioblastoma samples also revealed a mixture of transcriptomes characteristic of the proneural, neural, classical, and mesenchymal subtypes defined by The Cancer Genome Atlas (24, 25). However, the specific genomic and transcriptomic determinants that drive the development of epithelioid glioblastoma phenotype remain unclear. Second, the emergence of the epithelioid clone could be a result of the selection pressure exerted by the host's immune system. In our patient, monocytes and macrophages are the predominant immune cells in the glioblastoma component, while CD4+ lymphocytes and NK cells are found in the epithelioid component. Interestingly, epithelioid glioblastoma cells express MHC class II antigen, CD68, and colony-stimulating factor receptor-1 commonly found in microglia/macrophage lineage, and therefore, they may interact with CD4+ and NK cells in the process (26). These cells also possess phagocytic activity and lysozyme, and they secrete a massive amount of IL-6 when stimulated in cell culture (26). Therefore, it is plausible that our patient's glioblastoma precursor possesses genomic and transcriptomic differences at the level of individual tumor cells, and the subsequent infiltration of CD4+ and NK cells, which we believe is a stochastic process, provided selection pressure and promoted the emergence of the epithelioid glioblastoma phenotype.

Over half of the cases of epithelioid glioblastoma described in the literature harbor the *BRAF V600E*. In Wang's series of 33 patients, it is notable that the median age was 36 years, with a range from 9 to 67 years, and all had the *BRAF V600E* mutation (7). In another series of 24 cases with ages ranging from 3 to 54 years, Chatterjee et al. demonstrated BRAF V600E expression by immunohistochemistry in only 12 of 23 patients (8). However, in Kleinschmidt-DeMasters' series, 12 of 13 patients had ages ranging from 10 to 50 years and 7 had the mutation; only one was 69 years of age and his tumor did not have the mutation (4). Our patient's age is 86 years. To the best of our knowledge, she is probably the oldest individual reported with epithelioid glioblastoma, and her tumor did not have the mutation. Therefore, we speculate that the activating *BRAF V600E* mutation may be less frequently found in patients older than the age of 60 years. Furthermore, the overall efficacy of V600E-directed targeted therapies is unknown. There are only anecdotal reports of response using dabrafenib with or without trametinib (27–29). A patient was treated with single-agent vemurafenib at the time of recurrence and his disease was stabilized for at least 4 months (30). But the median duration of response in this population is uncertain. We speculate that despite possessing the *BRAF V600E* mutation, older patients may not respond to V600E-directed targeted therapy, probably due to the accumulation of additional somatic driver mutations or faster clonal evolution that promotes the emergence of resistant clones. Regardless, in our patient, *BRAF V600E* mutation is not present in her tumor, and therefore, the probability of response to targeted therapy is very low.

## Data availability statement

The raw data supporting the conclusions of this article will be made available by the authors, without undue reservation.

## Ethics statement

Ethical review and approval was not required for the study on human participants in accordance with the local legislation and institutional requirements. The patients/participants provided their written informed consent to participate in this study. Written informed consent was obtained from the individual for the publication of any potentially identifiable images or data included in this article.

## Author contributions

MM and EW: study concept, design, and drafting of the manuscript. MM, MK, JD, DB, MC, ST, and EW: data collection. MM, MK, JD, HE, SS, DB, MC, WK, SY, ST, and EW: analysis and interpretation of data. All authors contributed to the article and approved the submitted version.
