# Supplementary material for: Dimorphic glioblastoma with glial and epithelioid phenotypes: Clonal evolution and immune selection
Source: Front Neurol. 2023 Jan 10;13:1017087. doi: 10.3389/fneur.2022.1017087 (PMC9871816; doi:10.3389/fneur.2022.1017087)
Supplement: Supplementary file 1 [file Table_1.docx]

**SUPPLEMENTARY TABLE 1:** Human Leukocyte Antigen (HLA) typing of the glioma and epithelioid sections by whole transcriptome sequencing (WTS) and whole exome sequencing (WES).

| **Glioblastoma Section** | | |
| --- | --- | --- |
| **Gene** | **HLA Type by WTS** | **HLA Type by WES** |
| HLA-A | A*03:01:01,A*02:01:01 | A*02:01:126,A*03:01:01 |
| HLA-B | B*15:01:01,B*51:01:01 | B*15:01:01,B*51:01:63 |
| HLA-C | C*03:04:01,C*15:02:01 | C*15:02:01,C*03:03:29 |
| HLA-DPA1 | DPA1*01:03:01 | DPA1*01:03:01 |
| HLA-DPB1 | DPB1*04:01:01,DPB1*16:01:01 | DPB1*04:01:01,DPB1*16:01:01 |
| HLA-DQA1 | DQA1*03:01:01,DQA1*03:02:01 | DQA1*04:01:02,DQA1*03:01:01 |
| HLA-DQB1 | DQB1*03:91Q,DQB1*03:02:01 | DQB1*03:02:01,DQB1*03:96 |
| HLA-DRB1 | DRB1*04:01:01,DRB1*09:01:02 | DRB1*04:01:01,DRB1*09:01:02 |
| **Epithelioid Section** | | |
| **Gene** | **HLA Type by WTS** | **HLA Type by WES** |
| HLA-A | A*03:01:01,A*02:01:01 | A*02:01:126,A*03:01:01 |
| HLA-B | B*15:01:01,B*51:01:01 | B*15:01:01,B*51:01:58 |
| HLA-C | C*15:02:01,C*03:04:01 | C*15:02:01,C*03:04:01 |
| HLA-DPA1 | DPA1*01:03:01 | DPA1*01:03:01 |
| HLA-DPB1 | DPB1*04:01:01,DPB1*16:01:01 | DPB1*04:01:01,DPB1*16:01:01 |
| HLA-DQA1 | DQA1*03:01:01,DQA1*03:02:01 | DQA1*04:01:02,DQA1*03:01:01 |
| HLA-DQB1 | Indeterminate | DQB1*03:02:01,DQB1*03:96 |
| HLA-DRB1 | DRB1*04:01:01,DRB1*09:01:02 | DRB1*04:01:01,DRB1*09:01:02 |
